# Use of Electronic and Conventional Cigarettes and Self-Rated Mental Health in High School Students

**DOI:** 10.3390/children12070902

**Published:** 2025-07-08

**Authors:** Payam Sheikhattari, Rifath Ara Alam Barsha, Chidubem Egboluche, Shervin Assari

**Affiliations:** 1 School of Community Health and Policy, Morgan State University, Baltimore, MD 21251, USA; payam.sheikhattari@morgan.edu (P.S.); chegb4@morgan.edu (C.E.); 2 John D. Bower School of Population Health, University of Mississippi Medical Center, Jackson, MS 39216, USA; rbarsha@umc.edu; 3 Department of Internal Medicine, Charles R Drew University of Medicine and Science, Los Angeles, CA 90059, USA

**Keywords:** adolescents, tobacco use, e-cigarettes, mental health, urban youth, health disparities, Baltimore City, cross-sectional study, self-rated health, socioeconomic factors

## Abstract

**Background:** Youth tobacco use remains a significant public health concern, particularly in urban communities disproportionately burdened by health disparities. In Baltimore City, where tobacco-related harms are elevated, understanding the relationship between tobacco use—including e-cigarettes—and mental health among high school students is essential for guiding equitable prevention and cessation strategies. The CEASE (Communities Engaged and Advocating for a Smoke-free Environment) program, in collaboration with the American Lung Association’s Not On Tobacco (N-O-T) initiative, developed an online school-based survey to inform community-responsive interventions. **Aims:** This study aimed to examine the associations between cigarette use, including conventional cigarette use, and self-rated mental health among high school students in Baltimore City. **Methods:** High school students in Baltimore City completed an anonymous online survey that assessed demographic characteristics, tobacco knowledge and use, mental health, and related behaviors. Self-rated mental health was dichotomized as poor versus fair/good. Tobacco use categories included current use of e-cigarettes and conventional cigarettes. Logistic regression models were used to examine associations between tobacco use and self-rated mental health, adjusting for age, gender, race, and parental education. Odds ratios (ORs) with 95% confidence intervals (CIs) were reported. **Results:** No statistically significant associations were found between self-rated mental health and e-cigarette use and conventional tobacco use after adjusting for covariates. **Conclusions:** The absence of a significant association may reflect unique aspects of the social context in Baltimore City, where youth may not use tobacco products as a coping mechanism for mental health challenges. Alternatively, it may be due to limitations in measurement, particularly the use of a single-item mental health assessment. These findings should be considered preliminary. Future research using more comprehensive mental health measures and larger samples is warranted to further explore these complex relationships.

## 1. Introduction

The use of electronic cigarettes (e-cigarettes or “vapes”) has surged among adolescents in recent years [1], prompting widespread public health concern [2]. Initially introduced as a potentially safer alternative to combustible tobacco products [3], e-cigarettes have rapidly gained popularity among youth—many of whom have no prior history of conventional cigarette smoking [4,5]. In many urban regions across the United States (e.g., Baltimore), e-cigarette use has surpassed traditional cigarette smoking among high school students, marking a substantial shift in the pattern of adolescent nicotine consumption [6,7].

This growing prevalence of e-cigarette use among adolescents coincides with increased national attention to youth mental health [8,9,10]. Recent data suggest that a significant proportion of adolescents report psychological distress [10,11], including symptoms of anxiety, depression, and low mood. Several studies have noted that these mental health concerns may be more frequently reported among youth who use nicotine products [12,13]. However, the directionality and underlying mechanisms of this association remain unclear [14,15]. It is uncertain whether e-cigarette use contributes to poorer mental health, whether it reflects pre-existing psychological challenges, or whether both are shaped by shared contextual or behavioral risk factors [16,17].

Although some prior research has reported associations between e-cigarette use and adverse mental health outcomes in youth [18,19,20,21], relatively few studies have fully accounted for conventional cigarette use—a known correlate of psychological distress [22,23,24,25]. Given that e-cigarettes and combustible cigarettes are often co-used, failing to adjust for conventional smoking may confound estimates of the independent link between e-cigarette use and mental health. To better understand this relationship, it is important to separate the effects of each product, especially in urban settings where co-use and the clustering of risk exposures are common.

This study aimed to examine the associations between cigarette use, including conventional cigarette use, and self-rated mental health among high school students in Baltimore City, an urban setting with notable socioeconomic and health disparities. Findings from this work may inform school-based screening, prevention efforts, and policies addressing adolescent nicotine use and mental health in urban communities.

## 2. Methods

### 2.1. Design and Setting

This cross-sectional study utilized data from the CEASE Youth: School Survey, an online questionnaire administered to high school students in Baltimore City. The survey was developed as part of a school-based collaboration between Morgan State University’s CEASE program and the American Lung Association’s Not On Tobacco (N-O-T) initiative. The survey was conducted in participating public high schools across Baltimore and was designed to assess tobacco use, mental health, and related behavioral and demographic factors. Data collection occurred in a confidential, self-administered format, allowing for broad participation while minimizing response bias.

### 2.2. CEASE Youth

The CEASE Youth Survey design and administration is briefly explained here. The self-administered survey was delivered electronically and began with informed consent and the entry of basic, non-identifiable information, including the current date, the name of the school, a four-character respondent code based on their initials and phone number, and the month and year of birth. To preserve participant confidentiality, no names or directly identifying data were collected. Upon completion, each student received a ten-dollar electronic gift card as a token of appreciation.

### 2.3. Measures

The questionnaire included several core thematic areas. Participants were asked to report their age, gender identity, racial and ethnic background, current grade level in school, and perceived academic performance over the past year. Additional items captured household composition, including whether the respondent lived in a single-parent home, as well as parent or guardian education level and employment status. Household income was reported using categorical ranges. Respondents also indicated whether they had heard of various tobacco and nicotine products, including cigarettes, cigars, smokeless tobacco, hookah, and e-cigarettes, and answered a series of belief and attitude statements regarding the health risks, addictiveness, and chemical contents of these products, using a four-point Likert scale ranging from strongly agree to strongly disagree. The survey included items measuring lifetime and recent use of e-cigarettes and other tobacco products, asking participants whether they had ever used each product type, even once or twice. Additional items assessed secondhand smoke exposure within the home, in the community, and around the school environment. Participants were also asked whether they had ever consumed alcohol, including a few sips, or used marijuana, in order to assess patterns of co-occurring health risk behaviors. Students indicated whether they were aware of any tobacco cessation programs or resources offered by their school or community, with response options including yes, no, and not sure. Participants reported the frequency with which they encountered tobacco advertisements or promotions across various media platforms, including social media, streaming services, retail locations, and printed materials, and whether they had received any coupons or discount offers for tobacco products within the past year. The survey also captured technology use, with students identifying which digital platforms they used regularly for communication, including social media, messaging services, and email. Finally, questions on physical and mental health asked students to rate their general health status on a four-category scale (excellent, good, fair, or poor), and to report on cognitive difficulties and the frequency of mental health distress—including stress, anxiety, and depression—over the past thirty days. Participants also indicated whether a healthcare provider had advised them to stop using tobacco, including e-cigarettes, within the past year.

### 2.4. Data Use and Confidentiality

All survey data were stored and analyzed in a de-identified format. Results are reported in aggregate and will be used to evaluate the CEASE-NOT program and inform the development of targeted, evidence-based interventions aimed at reducing tobacco use among Baltimore City youth.

### 2.5. Institutional Review Board (IRB)

The study received ethical approval from the appropriate Institutional Review Board. All participants provided informed consent before beginning the survey. Parents approved the participation of their children in a consent form.

### 2.6. Study Variables

Self-Rated Mental Health. Mental health was assessed using a five-category item reflecting the number of days in the past month when mental health was “not good.” Response options included: *never*, *rarely*, *sometimes*, *most of the time*, and *always*. Participants who responded *most of the time* or *always* were categorized as having poor mental health, while all others were categorized as having fair/good mental health. For the purposes of this analysis, the variable was treated as dichotomous (0 = fair/good, 1 = poor).

Current Conventional Cigarette Use: Defined by a single item asking whether the respondent had smoked a conventional cigarette in the past 30 days.

Current E-Cigarette Use: Defined by a single item asking whether the respondent had used an e-cigarette in the past 30 days.

Covariates: Included participant age, gender, racial identity, and parent or guardian educational attainment.

### 2.7. Statistical Analysis

Descriptive statistics were generated to summarize the sample characteristics and distribution of primary variables. A series of logistic regression models were estimated, with self-rated poor or fair mental health as the dependent variable. Model 1 presents the unadjusted results of the independent variables, while Models 2 through 4 reflect sequentially adjusted models. In Model 2, demographic covariates (age, gender, and race) were added alongside current e-cigarette use. Model 3 included a socioeconomic variable (parent or guardian’s educational attainment) in addition to the variables from Model 2. Finally, Model 4 incorporated current conventional cigarette use along with all variables included in Model 3. Results are presented as odds ratios (ORs) with corresponding 95% confidence intervals (CIs). All analyses were conducted using Stata version 15.0.

## 3. Results

The descriptive characteristics of the study sample (*n* = 603) are presented in Table 1. The mean age of participants was 16.1 years (SD = 1.5). The majority of students were female (53.7%) and identified as Black (88.1%). Approximately 9.8% reported current e-cigarette use, and 6.1% reported current conventional cigarette use. Poor mental health was reported by 21.7% of the students.

Table 2 presents the bivariate correlations among the study variables. Male gender was significantly negatively correlated with poor mental health (r = −0.09, *p* < 0.05). No other variables showed a significant correlation with poor mental health.

The results of the unadjusted and adjusted logistic regression analyses are presented in Table 3. Model 1 presents the unadjusted results, while Models 2 through 4 reflect sequentially adjusted models. Male gender was consistently associated with significantly lower odds of poor mental health across all models (Model 1: OR = 0.63, *p* < 0.05; Model 2: OR = 0.61, *p* < 0.05; Model 3: OR = 0.62, *p* < 0.05; Model 4: OR = 0.62, *p* < 0.05). No statistically significant association was found between current e-cigarette use and mental health.

Table 4 presents the unadjusted and adjusted logistic regression results for poor mental health, stratified by gender. Among female participants, current e-cigarette use was consistently associated with higher odds of reporting poor mental health across all models. Although none of the associations reached statistical significance, the strength of the association increased in the fully adjusted model (Model 4: OR = 2.13, *p* = 0.09). Among male participants, no statistical significance was found between study variables and mental health.

Table 5 presents the unadjusted and adjusted logistic regression results for poor mental health, stratified by age group (≤16 years and >16 years). Among participants aged 16 years or younger, current e-cigarette use was significantly associated with higher odds of reporting poor mental health in the unadjusted model (Model 1: OR = 2.16, 95% *p* < 0.05). Although the association was no longer statistically significant in the adjusted models (Models 2–4), the elevated odds remained relatively stable (Model 4: OR = 2.04, *p* = 0.09). Among participants aged over 16 years, none of the predictors, including current e-cigarette use, were significantly associated with poor mental health in either unadjusted or adjusted models.

## 4. Discussion

This study aimed to examine the associations between cigarette use, including conventional cigarette use, and self-rated mental health among high school students in Baltimore City. Our findings revealed no statistically significant associations between self-rated mental health and the use of e-cigarettes and conventional cigarettes in this urban context. Even after adjusting for demographic covariates, tobacco use was not predictive of poor/fair mental health status in this sample.

Only among participants aged 16 years or younger, and only in unadjusted models, current e-cigarette use was significantly associated with higher odds of reporting poor mental health in the unadjusted model. This association did not remain significant in adjusted models. Similarly, among participants aged over 16 years, current e-cigarette use was not significantly associated with poor mental health in either unadjusted or adjusted models. Thus, age was not a moderator of our association of interest. Gender also did not moderate our association of interest. No statistical significance was found between e-cig use and mental health for males or females. These additional analyses showed that in our sample, e-cig use and mental health do not show an association.

To explain our observed null findings, it is important to consider the unique social and environmental context of Baltimore City. In predominantly low-income, urban communities marked by high levels of environmental stressors and structural disadvantage, and in line with the minorities’ diminished returns theory [26], the relationship between tobacco use and mental health may differ from national patterns and may be more difficult to detect. In such settings, youth may engage with tobacco products for reasons unrelated to emotional distress—such as peer influence, community norms, or widespread product availability [27,28]. Prior research suggests that in contexts where trauma and stress are highly prevalent, their effects on behaviors like tobacco use may be attenuated [29]. In other words, trauma may have a more pronounced influence on tobacco use when it is less common, while its impact may diminish in environments where it is widespread [30]. Another possibility is that youth in Baltimore may turn to alternative coping strategies rather than tobacco use when experiencing mental health challenges. Additionally, the underreporting of emotional distress due to stigma or limited access to mental health resources may obscure true levels of need [31,32]. Therefore, the absence of an observed association should not be interpreted as an absence of need but rather as an invitation to deepen contextual understanding.

Regardless of the underlying explanation, our null findings underscore the importance of conducting research that is sensitive to local context. Variations in social and structural determinants of health—such as poverty, neighborhood safety, school climate, and access to mental health services—can shape unique patterns in the relationship between tobacco use and mental health. Future studies should consider incorporating qualitative methods and longitudinal designs to better understand these complex interactions and to assess whether similar patterns are present in other urban settings.

In addition to our local analysis, national and state-level data from the CDC’s Youth Risk Behavior Survey (YRBS) also capture smoking, vaping, and mental health indicators among high school students [33,34,35,36]. For example, Monitoring the Future data [36] has provided extensive data across states. Also, Maryland’s combined YRBS/Youth Tobacco Survey (YTS) included nearly 60,000 public middle and high school students in 2022 and assessed tobacco use, vaping, and feelings of sadness or hopelessness, among other behaviors. While many states use YRBS data to guide policy, its scope is limited to state- or national-level estimates. As such, our findings offer a crucial, city-specific perspective: Baltimore City’s CEASE Youth Survey targets only local high school students and includes more granular questions on smoking, vaping, and self-rated mental health. Notably, Baltimore City has participated in the CDC’s YRBS, but participation for local-level (i.e., city-specific) representative YRBS data has varied over time. In contrast, CEASE Youth focuses explicitly on Baltimore, allowing us to examine associations at the city level that might be obscured in broader datasets. By situating our results alongside YRBS findings, we can contextualize the magnitude of tobacco-related and mental health issues in our unique local environment and underscore the value of city-level monitoring.

### 4.1. Limitations

This study has several important limitations that should be acknowledged when interpreting the findings. First, the cross-sectional design precludes any conclusions about causality or directionality. We cannot determine whether tobacco use contributes to mental health challenges, whether mental health difficulties lead to tobacco use, or whether both are influenced by shared underlying factors such as trauma or environmental stress.

Second, measurement limitations should be carefully considered when interpreting these findings. In this study, self-rated mental health was assessed using a single-item measure, drawn from a larger battery within the CEASE Youth questionnaire. This approach was selected to reduce participant burden; however, it may not fully capture the complexity of adolescent psychological well-being. Single-item measures may lack sensitivity and nuance, particularly in capturing subclinical or domain-specific symptoms such as depression, anxiety, or stress. It is possible that some participants who did not report poor mental health on this broad item may still experience significant psychological symptoms—such as sadness, worry, or irritability—when asked using more detailed, symptom-specific tools. Consequently, multi-item, validated instruments assessing various aspects of mental health could yield different patterns of association with tobacco use.

Third, all data were self-reported and, therefore, subject to recall bias and social desirability bias, particularly in a school setting where students may underreport sensitive behaviors or emotional struggles. Fourth, while the sample reflects students from an urban area with high health disparities, the findings may not be generalizable to adolescents in suburban, rural, or more socioeconomically diverse populations. Fifth, our measures of tobacco use focused on ever-use rather than frequency, recency, or dependence, which may obscure important distinctions in behavioral and psychological profiles among users. Sixth, due to the anonymous nature of the survey and limited space in the questionnaire, we were unable to assess other key variables that may confound or mediate the relationship between tobacco use and mental health, such as adverse childhood experiences, family tobacco use, school engagement, or peer influence. Finally, the survey was conducted within the framework of a community-engaged prevention initiative, which may have influenced student perceptions or responses. Despite these limitations, the study provides a critical foundation for more detailed, longitudinal research on the intersection of mental health and tobacco use in marginalized urban youth populations.

### 4.2. Direction for Future Research

Future studies should aim to use broader mental health assessments and consider additional factors such as trauma exposure, peer networks, and access to recreational alternatives. Exploring how race, gender, and structural inequalities intersect to shape tobacco behaviors and psychological health is also crucial, especially in under-resourced urban communities.

## 5. Conclusions

Although this study found no significant association between tobacco use and self-rated mental health among high school students in Baltimore City, the findings underscore the importance of context in shaping behavioral health outcomes. These preliminary results call for deeper investigation using more comprehensive mental health measures, diverse geographic settings, and longitudinal data. Understanding the nuanced relationship between mental health and tobacco use is essential to designing equitable, youth-centered prevention and cessation programs in urban communities.

## Figures and Tables

**Table 1:** Descriptive statistics of the study sample.

Variables	Total (*n* = 603)
Current E-cigarette Use	
No	544 (90.2)
Yes	59 (9.8)
Conventional Cigarette Use	
No	566 (93.9)
Yes	37 (6.1)
Mental Health	
Poor	131 (21.7)
Fair/Good	466 (77.3)
Missing	6 (1.00)
Gender	
Female	324 (53.7)
Male	260 (43.1)
Missing	19 (3.2)
Race	
Black	531 (88.1)
White	20 (3.3)
Others	46 (7.6)
Missing	6 (1.0)
Parent or Guardian’s Educational Attainment	
Less than high school diploma	74 (12.3)
High school graduation	214 (35.5)
Some college	129 (21.4)
College graduation	78 (12.9)
Graduate degree or higher	104 (17.2)
Missing	4 (0.7)
	Mean (SD)
Age (Years)	16.1 (1.5)

**Table 2:** Correlation matrix of the study variables.

Variables	1	2	3	4	5	6
1. Mental Health	1.00					
2. Current E-cigarette Use (Yes)	0.07	1.00				
3. Current Conventional Cigarette Use (Yes)	0.01	0.33 ***	1.00			
4. Age (Years)	0.03	−0.04	0.08	1.00		
5. Gender (Male)	−0.09 *	−0.06	−0.03	0.09 *	1.00	
6. Parent or Guardian’s Educational Attainment	0.02	−0.12 **	−0.08 *	−0.08	0.03	1.00

Note. * *p* < 0.05, ** *p* < 0.01, *** *p* < 0.001.

**Table 3:** Logistic regression results, overall.

Variables	Poor Mental Health			
	Unadjusted OR (95% CI)	Adjusted OR (95% CI)		
	Model 1	Model 2	Model 3	Model 4
Current E-cigarette Use				
No	Ref.	Ref.	Ref.	Ref.
Yes	1.70 (0.94, 3.07)	1.67 (0.90, 3.08)	1.68 (0.90, 3.13)	1.67 (0.86, 3.25)
Age	1.05 (0.92, 1.19)	1.06 (0.92, 1.22)	1.07 (0.93, 1.23)	1.07 (0.93, 1.23)
Gender				
Female	Ref.	Ref.	Ref.	Ref.
Male	0.63 * (0.42, 0.95)	0.61 * (0.40, 0.93)	0.62 * (0.41, 0.95)	0.62 * (0.41, 0.95)
Race				
White	Ref.	Ref.	Ref.	Ref.
Black	2.51 (0.57, 10.99)	5.22 (0.69, 39.77)	5.33 (0.70, 40.62)	5.34 (0.70, 40.81)
Others	3.66 (0.74, 18.05)	7.49 (0.89, 62.96)	7.64 (0.91, 64.42)	7.66 (0.91, 64.76)
Parent or Guardian’s Educational Attainment				
Less than high school diploma	Ref.	NA	Ref.	Ref.
High school graduation	1.08 (0.56, 2.09)	NA	1.15 (0.59, 2.26)	1.15 (0.58, 2.26)
Some college	1.17 (0.58, 2.36)	NA	1.34 (0.64, 2.78)	1.34 (0.64, 2.78)
College graduation	0.85 (0.38, 1.90)	NA	0.85 (0.36, 2.02)	0.85 (0.36, 2.02)
Graduate degree or higher	1.29 (0.63, 2.65)	NA	1.38 (0.65, 2.93)	1.38 (0.65, 2.93)
Current Conventional Cigarette Use				
No	Ref.	NA	NA	Ref.
Yes	1.15 (0.53, 2.51)	NA	NA	1.02 (0.43, 2.40)

Note. * *p* < 0.05. Abbreviations: OR = odds ratio, CI = confidence interval.

**Table 4:** Logistic regression results stratified by gender.

Variables	Poor Mental Health			
	Unadjusted OR (95% CI)	Adjusted OR (95% CI)		
	Model 1	Model 2	Model 3	Model 4
**Female**				
Current E-cigarette Use				
No	Ref.	Ref.	Ref.	Ref.
Yes	1.63 (0.77, 3.43)	1.66 (0.78, 3.52)	1.81 (0.83, 3.92)	2.13 (0.88, 5.13)
Age	1.02 (0.86, 1.22)	1.03 (0.86, 1.23)	1.07 (0.89, 1.28)	1.07 (0.89, 1.29)
Race				
White	Ref.	Ref.	Ref.	Ref.
Black	3.27 (0.41, 26.01)	3.42 (0.43, 27.31)	3.73 (0.47, 29.97)	3.58 (0.44, 28.85)
Others	5.00 (0.54, 46.22)	5.21 (0.56, 48.41)	5.96 (0.63, 56.07)	5.65 (0.60, 53.46)
Parent or Guardian’s Educational Attainment				
Less than high school diploma	Ref.	Ref.	Ref.	Ref.
High school graduation	0.89 (0.39, 2.01)	NA	0.95 (0.42, 2.19)	0.97 (0.42, 2.24)
Some college	1.54 (0.66, 3.55)	NA	1.86 (0.77, 4.45)	1.88 (0.78, 4.53)
College graduation	0.76 (0.26, 2.21)	NA	0.90 (0.31, 2.68)	0.92 (0.31, 2.73)
Graduate degree or higher	1.17 (0.48, 2.86)	NA	1.38 (0.55, 3.48)	1.38 (0.54, 3.48)
Current Conventional Cigarette Use				
No	Ref.	Ref.	Ref.	Ref.
Yes	0.89 (0.32, 2.51)	NA	NA	0.63 (0.19, 2.12)
**Male**				
Current E-cigarette Use				
No	Ref.	Ref.	Ref.	Ref.
Yes	1.59 (0.55, 4.59)	1.67 (0.57, 4.88)	1.61 (0.54, 4.81)	1.55 (0.51, 4.66)
Age	1.11 (0.89, 1.40)	1.12 (0.89, 1.41)	1.12 (0.88, 1.41)	1.09 (0.86, 1.39)
Race				
White	Ref.	Ref.	Ref.	Ref.
Black	0.77 (0.24, 2.46)	0.76 (0.24, 2.44)	0.89 (0.27, 2.92)	0.90 (0.27, 2.95)
Others	Omitted	Omitted	Omitted	Omitted
Parent or Guardian’s Educational Attainment				
Less than high school diploma	Ref.	Ref.	Ref.	Ref.
High school graduation	1.47 (0.45, 4.74)	NA	1.35 (0.40, 4.53)	1.28 (0.38, 4.32)
Some college	0.72 (0.18, 2.80)	NA	0.57 (0.13, 2.43)	0.55 (0.13, 2.33)
College graduation	0.81 (0.20, 3.35)	NA	0.71 (0.16, 3.08)	0.70 (0.16, 3.05)
Graduate degree or higher	1.46 (0.40, 5.31)	NA	1.32 (0.35, 4.95)	1.28 (0.34, 4.80)
Current Conventional Cigarette Use				
No	Ref.	Ref.	Ref.	Ref.
Yes	2.04 (0.61, 6.83)	NA	NA	1.81 (0.49, 6.65)

Abbreviations: OR = odds ratio, CI = confidence interval.

**Table 5:** Logistic regression results stratified by age group.

Variables	Poor Mental Health			
	Unadjusted OR (95% CI)	Adjusted OR (95% CI)		
	Model 1	Model 2	Model 3	Model 4
**Age ≤ 16**				
Current E-cigarette Use				
No	Ref.	Ref.	Ref.	Ref.
Yes	2.16 * (1.05, 4.44)	2.00 (0.94, 4.25)	2.01 (0.94, 4.33)	2.04 (0.89, 4.68)
Gender				
Female	Ref.	Ref.	Ref.	Ref.
Male	0.62 (0.35, 1.08)	0.62 (0.35, 1.09)	0.64 (0.36, 1.13)	0.64 (0.36, 1.13)
Race				
White	Ref.	Ref.	Ref.	Ref.
Black	2.46 (0.31, 19.58)	0.64 (0.27, 1.54)	0.64 (0.27, 1.55)	0.64 (0.27, 1.55)
Others	3.64 (0.40, 33.12)	Omitted	Omitted	Omitted
Parent or Guardian’s Educational Attainment				
Less than high school diploma	Ref.	Ref.	Ref.	Ref.
High school graduation	0.75 (0.32, 1.74)	NA	0.75 (0.31, 1.78)	0.75 (0.31, 1.82)
Some college	0.78 (0.32, 1.90)	NA	0.83 (0.33, 2.09)	0.83 (0.33, 2.11)
College graduation	0.68 (0.23, 2.00)	NA	0.75 (0.25, 2.24)	0.75 (0.25, 2.26)
Graduate degree or higher	1.02 (0.41, 2.50)	NA	1.02 (0.40, 2.63)	1.02 (0.40, 2.65)
Current Conventional Cigarette Use				
No	Ref.	Ref.	Ref.	Ref.
Yes	1.16 (0.37, 3.65)	NA	NA	0.95 (0.25, 3.57)
**Age < 16**				
Current E-cigarette Use				
No	Ref.	Ref.	Ref.	Ref.
Yes	1.16 (0.40, 3.40)	1.28 (0.43, 3.80)	1.39 (0.45, 4.27)	1.33 (0.41, 4.27)
Gender				
Female	Ref.	Ref.	Ref.	Ref.
Male	0.62 (0.34, 1.14)	0.60 (0.32, 1.11)	0.58 (0.31, 1.10)	0.58 (0.31, 1.10)
Race				
White	Ref.	Ref.	Ref.	Ref.
Black	2.65 (0.32, 21.68)	2.47 (0.30, 20.40)	2.46 (0.29, 20.64)	2.46 (0.29, 20.59)
Others	4.00 (0.39, 41.51)	3.17 (0.29, 34.58)	3.26 (0.29, 36.84)	3.26 (0.29, 36.78)
Parent or Guardian’s Educational Attainment				
Less than high school diploma	Ref.	Ref.	Ref.	Ref.
High school graduation	1.79 (0.62, 5.21)	NA	1.98 (0.65, 6.00)	1.98 (0.65, 6.04)
Some college	2.26 (0.72, 7.12)	NA	2.71 (0.81, 9.02)	2.72 (0.81, 9.06)
College graduation	1.17 (0.33, 4.13)	NA	1.08 (0.27, 4.40)	1.09 (0.27, 4.44)
Graduate degree or higher	1.92 (0.58, 6.42)	NA	2.17 (0.63, 7.45)	2.20 (0.64, 7.55)
Current Conventional Cigarette Use				
No	Ref.	Ref.	Ref.	Ref.
Yes	1.07 (0.37, 3.11)	NA	NA	1.18 (0.38, 3.70)

*Note.* * *p* < 0.05. Abbreviations: OR = odds ratio, CI = confidence interval.

## Data Availability

The original contributions presented in this study are included in the article. Further inquiries can be directed to the corresponding author.
